# Ultrasound synthetic aperture non-line-of-sight imaging

**DOI:** 10.1038/s42005-025-02335-3

**Published:** 2025-11-17

**Authors:** Tailin Li, Ilya Starshynov, Khaled Kassem, Zongliang Xie, Ge Ren, Yihan Luo, Daniele Faccio

**Affiliations:** 1 National Key Laboratory of Optical Field Manipulation Science and Technology, Chinese Academy of Science, Chengdu, China; 2 Key Laboratory of Optical Engineering, Chinese Academy of Science, Chengdu, China; 3 Institute of Optics and Electronics, Chinese Academy of Science, Chengdu, China; 4 University of Chinese Academy of Science, Beijing, China; 5 School of Physics & Astronomy, University of Glasgow, Glasgow, UK

**Keywords:** Acoustics, Imaging and sensing

## Abstract

Non-line-of-sight (NLOS) imaging typically relies on the use of ultrashort laser pulses and time-resolved detection to then reconstruct 3D environments that are hidden from the direct line-of-sight. However, the same scattering mechanism and wall-reflections that allow light to propagate into the hidden environment and back again ultimately limit both resolution and imaging distances even at high laser powers. Non-optical, such as acoustic and radio-wave approaches promise to solve some of these issues but have yet to achieve results comparable to optical systems. We present an ultrasound-based NLOS imaging system based on a scanning ultrasound emitter and receiver operating in a frequency range similar to common bats that demonstrates high-resolution 3D reconstruction of hidden scenes. We successfully image multiple targets and complex scenes with  ~ cm depth resolution at distances up to 2 m away from the scattering surface. Measurements of the NLOS modulation transfer function quantify the spatial resolution to also be  ~ 1 cm, which is comparable to traditional optical NLOS techniques.

## Abstract (web summary)

Optical non-line-of-sight imaging demands ultrafast detectors and suffers steep power loss, limiting range and practicality. Here, the authors realize a phase-sensitive synthetic-aperture ultrasound platform that exploits specular wall reflections and f-k migration to reconstruct 3D hidden scenes with 1 cm lateral resolution at meter-scale ranges, attaining performance comparable to optical approaches with low-power, eye-safe hardware.

## Introduction

In recent years, significant efforts have been devoted to recovering scenes hidden e.g., by a wall, by analyzing the return scattered light using techniques commonly known as non-line-of-sight (NLOS) imaging^1,2^. These techniques have a range of potential applications for autonomous driving, emergency rescue, medical imaging, etc. Currently, most of the existing NLOS imaging methods are based on optical time-resolved systems, e.g., laser sources that emit picosecond pulses onto a relay surface and a synchronized single-photon detector with a time-correlated single photon counting (TCSPC) module that records the travel time of the returning photons^3–8^. Despite the notable success of existing optical methods that can now image human-sized scenes 1–3 m from the relay surface, optical NLOS imaging still faces some challenges. The imaging distance behind a wall is limited by the rapid fourth-power light intensity decay with distance. In order to partly compensate for the reduction in return photon numbers, high-power (e.g., 1 W or higher) lasers are required, which may pose potential risks to eye safety. Passive imaging techniques of course, would resolve these challenges but have only been demonstrated to operate at limited imaging distances and/or require specific imaging conditions to be met (such as the presence of an additional obstacle or use of a corner) and can have limited spatial resolution^9–11^. Methods based on speckle correlometry^12–14^ or synthetic wave holography^15^ can achieve high resolution, but they suffer from practical constraints, such as limited field of view, stringent interferometric stability requirements and ambiguous phase retrieval reconstruction that might limit their usability.

Non-optical methods for NLOS imaging have also been demonstrated. The initial attempts at acoustic NLOS imaging involved experiments using a frequency-modulated continuous wave (FMCW) method to estimate the distance to hidden targets using a 2–20 kHz frequency sweep signal^16^. Further development of the acoustic approach introduced neural networks (ANNs) for the final image reconstruction^17,18^. Acoustic localization studies for hidden objects have also been performed^19–21^. Compared to optical NLOS imaging, there are several advantages of using ultrasound. At ultrasound wavelengths most common surfaces are acoustically smooth, so reflections off both the relay wall and the hidden object are predominantly specular. As a result, the back-scattered ultrasound suffers only the two-way geometric spreading loss-i.e., an intensity decay scaling as 1/*r*^2^ over the transceiver-wall-object path. This quadratic-versus-quartic decay can yield a substantial signal-to-noise advantage for ultrasound NLOS in many practical scenarios. Moreover, the use of ultrasound, of course, also removes any concerns regarding eye safety.

Finally, millimeter-wave^22^ and terahertz-wave^23^ electromagnetic radiation have been used for NLOS imaging. However, despite these advances, non-optical NLOS imaging has remained impractical owing to the inability to achieve comparable results with optical systems.

In this work, we demonstrate high-resolution ultrasound NLOS imaging. We conducted NLOS imaging experiments in various scenarios, utilizing the frequency-wavenumber (f-k) migration method for image reconstruction. The image reconstructions are shown to improve significantly when using the full acoustic field (as opposed to the pulse envelope used in the optical domain) and achieve  ~1 cm resolution at 1 m distances, demonstrating performance comparable to traditional time-of-flight optical methods. Our method is still able to distinctly detect and reconstruct the shape of objects at distances of 3 m from the transceiver (or 2 m from the relay wall). We believe that the combination of high resolution, low-cost components, and the enhancements outlined below paves the way for practical NLOS imaging systems.

## Results

### Ultrasound imaging model

**Fig. 1:**
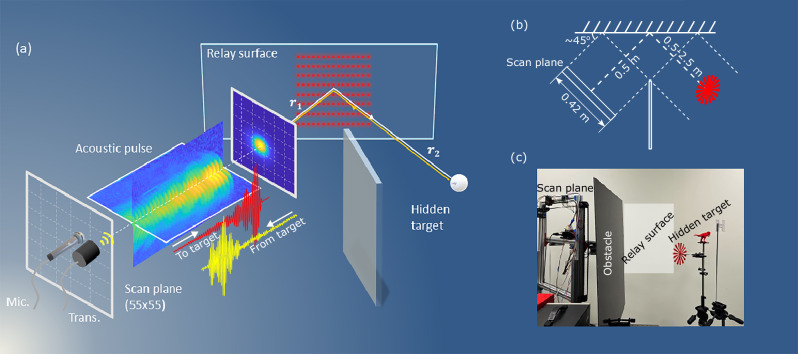
Overview of ultrasound NLOS imaging. **a** Schematic illustration of the experiments. A ultrasound transducer and a microphone are co-located and can be scanned at 55 × 55 points across a 42 × 42 cm area. The experimentally measured ultrasound pulse is shown, emitted from the transceiver towards the first relay surface (at distance *r*_1_, red dots indicate scan positions). This then reflects into the hidden scene area where (at distance *r*_2_) it is back-reflected from a hidden object and, after reflection from the relay wall, is measured by the microphone (typical acoustic waveforms before and after interacting with the scene are shown in red and yellow, respectively). **b** To-scale diagram of the experiment showing the relevant distances. **c** Shows a photograph of the experiment.

In most optical NLOS imaging schemes, the ability to spatially resolve hidden objects is achieved by scanning a relay scattering surface with a pulsed light beam. This Lambertian scattering surface redirects the focused laser beam into hemispherical wavefronts that hit the hidden scene which is then reconstructed from the temporal traces of the return signal at different illumination or observation points on the first relay wall. In contrast to optical NLOS imaging, focusing the sound wave into a narrow beam on the relay surface is challenging. However, differently from light, most flat surfaces are mirror-like for sound, which essentially equates line-of-sight (LOS) and NLOS scenarios. We then rely on the fact that synthetic apertures can be used for imaging when narrow focusing is not possible. Therefore, we utilize this approach to achieve spatial resolution. Figure 1a, illustrates our experimental setup (see also Methods) and a photograph is shown in Fig. 1b. An ultrasound (50 or 100 kHz) air transducer is used to send short-duration (~300 μs) ultrasound pulses onto the scene. The return signal is detected by an externally polarized condenser microphone. Both the transducer and the microphone are placed on a 2-axis scanning stage with a total range of motion of 42 cm in both vertical and horizontal dimensions. The divergence angle of the transducer is such that it creates  ~50 cm diameter sound pressure area at 1.6 m distance. Spatial information from the scene is therefore significantly blurred and would be limited only to large  ~1 m size objects. The return signal measured by the microphone (for the direct line-of-sight case) at a position $({x}^{{\prime} },{y}^{{\prime} })$ at the scanning plane can be expressed as a time-varying interference pattern *τ* that encodes the spatial information about the scene:

1 $$\tau ({x}^{{\prime} },{y}^{{\prime} },t) = \iint \!\!\int \frac{1}{{(2r)}^{\alpha }}\cdot \rho (x,y,z)\cdot \\ E({x}^{{\prime} }-x,{y}^{{\prime} }-y,z-vt,t)dxdydz$$

where $\tau ({x}^{{\prime} },{y}^{{\prime} },t)$ is the sampled acoustic pressure at a point $({x}^{{\prime} },{y}^{{\prime} })$, *E* is the pressure field created by the transducer, *ρ* is the spatial scattering (reflectivity) distribuion of the scene, *v* is the speed of sound, $r=\sqrt{{x}^{2}+{y}^{2}+{z}^{2}}$ and the parameter *α*, (0 < *α* < 4), depends on the scattering properties of the wall and the objects within the scene (*α* = 0 for fully specular reflection and *α* = 4 for a fully diffusive reflection from both the relay wall and the object). We experimentally evaluated *α* = 1.6 and 2.1 for an acoustic wave at 50 kHz and 100 kHz, respectively (see “Methods”).

Since most of the developed methods for NLOS image reconstruction, such as backprojection, linear inverse (light-cone) and inverse light transport rely on the assumption of Lambertian scattering from the first relay wall, these are not directly applicable in this scenario^1^. In contrast, wave-based methods are not affected by this limitation. Moreover, these can effectively handle the wave-like behavior of the sound pressure recorded in the detection plane. We therefore adapt the f-k migration method to reconstruct the hidden scene. In exploration seismology, migration refers to a multi-channel processing step that attempts to spatially re-position seismic events and improve focusing^24^, which is similar to the imaging problem considered here. The f-k migration method is a Fourier-based-approach applied to migration problems. Proposed by Stolt in 1978, it is based on an exact solution to the wave equation and thus is able to model wave reflection, scattering and other complex propagation scenarios, which is also suitable for dealing with specular reflection in the ultrasound domain. Furthermore, it is the fastest known migration technique.

In f-k migration, the problem statement is the following: given the solution to the wave equation *Ψ*(*x*, *y*, *z* = 0, *t*) at a particular plane (here taken to be *z* = 0), find the initial condition *Ψ*(*x*, *y*, *z*, *t* = 0) corresponding to that solution. In our case, the quantities of interest are the sound pressure *τ*(*x*, *y*, *z* = 0, *t*) and *τ*(*x*, *y*, *z*, *t* = 0)—finding the latter is equivalent to finding *ρ*(*x*, *y*, *z*). It has been shown that *Ψ*(*x*, *y*, *z* = 0, *t*) and *Ψ*(*x*, *y*, *z*, *t* = 0) are related through two Fourier transforms and an interpolation^5,24^. Therefore, in order to find *τ*(*x*, *y*, *z*, *t* = 0), we need to apply these three steps to *τ*(*x*, *y*, *z* = 0, *t*).

### Experimental results

**Fig. 2:**
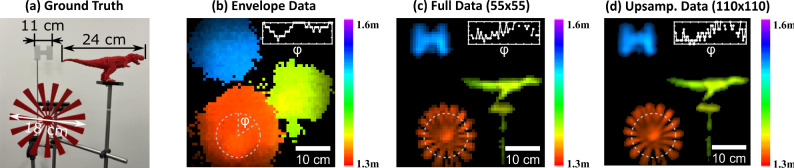
3D scene reconstruction. **a** Photograph of a scene containing three objects at different distances, as viewed from the scan plane. **b** Reconstruction of the scene using the signal envelope in the f-k migration algorithm. **c** Reconstruction using full phase information, revealing fine details such as the petals of the star. **d** Reconstruction with spatial interpolation of the raw data cube prior to f-k migration, further improving resolution. The insets in each panel provide reconstructed depth profiles along the dashed circles with the vertical axis matching the colorbar scale. The resolution target was circular with a diameter of 18 cm.

In optical NLOS imaging, the input to the f-k migration algorithm $\sqrt{\tau (x,y,t)}$, typically does not contain phase information, as only photon intensity and arrival time is detected. Here we record the sound pressure wave, which therefore contains also phase information and can lead to a richer reconstruction. We explicitly compare the results of the reconstruction with and without phase information (see “Methods”). Figure 2a shows a photograph of the scene that we image and that contains multiple objects of varying complexity. Figure 2b shows the reconstruction using only envelope (no phase) information. In contrast, Fig. 1c shows the reconstruction result for the same scene when the full waveform is used: the inclusion of phase information allows for the resolution of fine details in the object shapes that are missing in Fig. 2b.

In Fig. 2d, we improve the resolution further by first upsampling the raw 3D data cube by a factor of 2 × 2 using cubic spline interpolation and then applying the f-k migration algorithm, resulting in more accurate spatial details. It is well known that simple interpolation cannot add new details to an image, so we support this claim with a detailed investigation of the final image resolution.

**Fig. 3:**
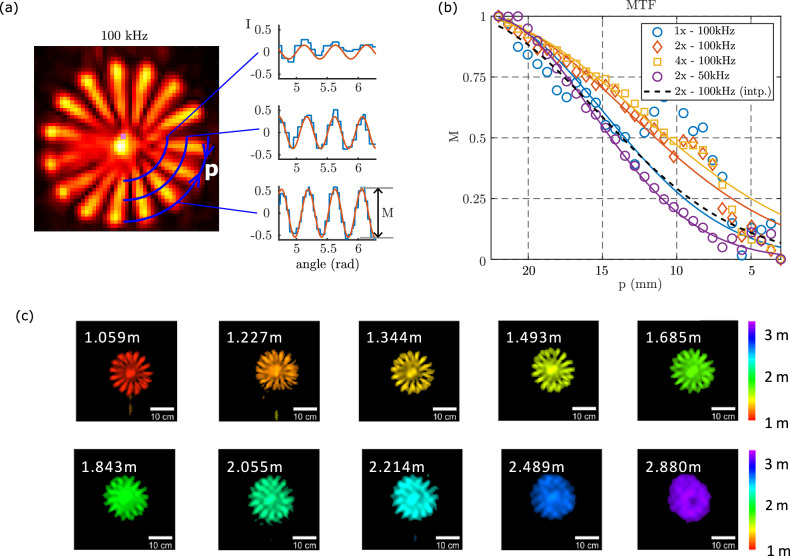
Lateral reconstruction resolution. **a** Modulation transfer function (MTF) estimation process, where intensity values along concentric circles are analyzed and fitted with sinusoidal functions. **b** Measured MTFs with error-weighted fits (solid lines) for 50 and 100 kHz at 1 m, comparing different upsampling rates (1× , 2× , and 4×) applied to a 55 × 55 raw measurement cube. Additionally, a conventional bicubic interpolation from a 55 × 55 reconstruction to 110 × 110 (black dashed) is shown, demonstrating no resolution improvement over the non-interpolated case. **c** Reconstruction results at distances of 1–3 m from the scan plane using 2 × 2 upsampled data. The individual petals of the object become indistinguishable beyond 2 m.

To experimentally estimate the resolution, which is rarely addressed in NLOS literature, we imaged a Siemens resolution star, see Fig. 3a for an example image taken at 1 m distance and 100 kHz ultrasound frequency. This is used to evaluate the modulation transfer function (MTF) of our NLOS imaging system^25^. Specifically, we measured the MTF by selecting the intensity values along the concentric circles with a varying radius and fitting sinusoidal functions to these curves, as shown in Fig. 3a. The lateral resolution (width of the point spread function) is quantified as the separation *p* between the star petals where the sinusoid amplitude drops to half its maximum value (50% contrast)^25^.

Figure 3b shows the MTFs at a distance of 1 m from the transducer scan plane using 100 kHz transducer for the cases of: no-upsampling of the raw-data before f-k migration (blue circles and curve); a 2x upsampling before f-k migration (red diamonds and curve); a 4x upsampling before f-k migration (yellow squares and curve); 2x upsampling *after* f-k migration (black dashed curve); 2x upsampling before f-k migration at 50 kHz (purple circles and curve). In all cases, the solid curves are weighted Gaussian fits to the actual data (symbols).

These results show that a maximum resolution of  ~10 mm is obtained when 2x upsampling before f-k migration and is also significantly improved compared to the  ~15 mm resolution obtained either with non-upsampled data or with upsampling performed on the final image *after* f-k migration. This verifies that indeed, simple interpolation of the final image does not add new information or features but that performing the interpolation on the raw data before the final image retrieval can improve resolution. However, this improvement is limited in the sense that a 4x interpolation does not lead to any further resolution enhancement compared to the 2x case. Finally, the same resolution enhancement is seen also at 50 kHz where a ~15 mm resolution is observed and that is similar to the native (not upsampled) 100 kHz resolution.

In Supplementary Note 1, we theoretically analyze the resolution of our approach. The *f*–*k* migration algorithm fully preserves the resolution compared to the line-of-sight scenario, with the primary limitation arising from the finite imaging aperture (scan area). Thus, the lateral resolution is *δx*/*y* = *λ*/2NA and the axial resolution is *δz* = *v*/2Δ*f*, where NA is the numerical aperture of the setup (see Supplementary Note 1), *λ* is the acoustic wavelength, *v* is the speed of sound and Δ*f* is the bandwidth of the acoustic pulse. At 1 m distance, the lateral resolution limits are 8.3 mm and 16.6 mm for 100 kHz and 50 kHz, respectively, which closely match our experimental performance.

**Fig. 4:**
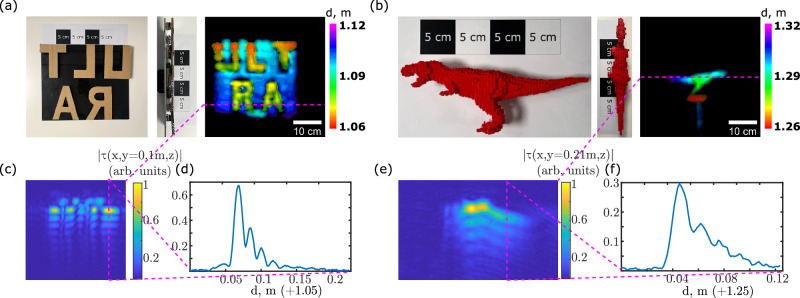
Depth resolution. **a** Ground truth of a board with the letters “ULTRA” shown from the front and top views, along with the corresponding reconstruction, highlighting depth variations on the board and letters. **b** Ground truth (front and top views) and reconstruction of a 3D-printed dinosaur. **c**, **e** Show the top views of the corresponding scenes at a plane indicated with dashed magenta lines. **d**, **f** show the cross-sections of (**c**) and (**e**), respectively, across the dashed magenta lines.

It is more challenging to practically estimate the axial resolution. The bandwidth of the 50 kHz peak in the pulse spectrum is around 5 kHz, which should lead to a 34 mm axial resolution. However, the pulse spectrum has a strong sideband at 60 kHz, which broadens the response and provides more depth information. Moreover, the irregular spectrum shape deteriorates the reconstruction process. The 100 kHz transducer shows a similar spectrum with an additional peak at 120 kHz, but the bandwidth is a bit larger, around 11 kHz. These sidebands could be eliminated by designing a transducer with an appropriately tailored frequency and temporal response. In Fig. 4, we show experimental reconstruction results that test the depth resolution. In Fig. 4a, cardboard letters are attached to an aluminum panel with an offset of 2 cm and are clearly distinguishable in the image. Furthermore, our imaging technique captures sub-centimeter distance gradients that are due to the slight inclination of the board with respect to the scan plane. In Fig. 4c, e, we show cross-sections (top views) of the two reconstructed scenes. Both exhibit ringing artifacts caused by the irregular pulse shape, which complicates the estimation of the axial resolution. We define the resolution as the FWHM of the strongest peaks in the axial profiles of these scenes, shown in Fig. 4d, f. This yields a resolution of 14 mm for both cases. Although this value is significantly larger than the diffraction-limited resolution achievable at Δ*f* = *f* (i.e., a single-period pulse), which corresponds to 3.4 mm, it remains substantially better than the estimate obtained from the measured Δ*f*. This indicates that the broader features of the spectrum indeed contribute additional information.

We note that, beyond acoustic approaches, such resolution is hard to achieve with time-of-flight-based optical techniques. The resolutions achieved here at 1 m from the scan plane would require an optical system with a time-of-arrival detector that has a resolution of 10 ps when using a similar experimental geometry (Supplementary Eqs. S6–S8), which is at the limits of current detector capabilities. While there have been reports of detectors with 10 ps or better resolution^3,26–28^, achieving such resolution in experiments remains a very challenging task. Also a number of techniques such as Synthetic Wave Holography^15^ or Speckle Correlation based techniques^12–14^ provide much better resolution (up to the optical diffraction limit) but they usually have certain limitations that can limit practical NLOS scenarios. In Supplementary Note 2, we present a comparison table (Supplementary Table 1) of various NLOS implementations and benchmark them against our approach, showing that we achieve resolutions comparable to those of optical techniques.

## Discussion

We have shown that ultrasound NLOS imaging can achieve diffraction-limited resolution. The combination of nearly specular reflections from surfaces (as opposed to diffusive reflections) and the ability to capture the full acoustic waveform—thus utilizing both amplitude and phase in image reconstruction—compensates for the longer wavelengths of acoustic waves compared to light-based systems. As a result, we demonstrate resolutions on the order of  ~1 cm at imaging distances of  ~1 m, which are comparable to many optical NLOS systems.

We acknowledge that certain optical techniques have achieved significantly better absolute spatial resolutions. In particular, methods employing coherent wavefront shaping or speckle memory effect reconstruction, synthetic wavelength holography or nonlinear optical upconversion with temporal resolutions approaching 1 ps^28^ can provide up to an order of magnitude improvement over ultrasound NLOS. We note, however, that these optical methods, while impressive in resolution, often involve more complex setups, require interferometric scene stability and have long (sometimes hours) acquisition times.

One possible limitation of our system is that the relay wall should be smooth compared to the acoustic wavelength, since centimeter-scale roughness would scramble the ultrasound similarly to how micron-scale roughness scrambles the light. However, even curved relay surfaces can yield useful reconstructions: although they introduce geometric distortions, these can often be corrected in post-processing to recover meaningful images. Optical NLoS techniques face the same requirement for relatively smooth relay surfaces, and they additionally fail in the presence of volumetric scattering (e.g., smoke or fog) because they rely on filtering the exponentially attenuated unscattered light. By contrast, airborne particulates at typical concentrations do not appreciably scatter 50–100 kHz ultrasound, so our acoustic NLoS approach remains effective in such challenging environments.

Our current system uses a single transducer on a linear mechanical stage which requires about 20 min for a 55 × 55 scan. Nearly all of this time is due to a delay at each scan point that is introduced in order to allow the system’s mechanical vibrations (resulting from the transuducer shifting) to settle down. Indeed, we underline that at each scan position we are acquiring in “single-shot”, differently from optical techniques that typically acquire over many laser pulse repetitions, we acquire just one single ultrasound pulse. Replacing the stage with a goniometric scanner or an electronically steered transducer array would remove all “settling” delays. Given that the round-trip travel time for a sound pulse to a 3 m target is only 17 ms, without any mechanical pauses a full scan could therefore be completed in  ~50 s. The acquisition speed could be further reduced by adding frequency-multiplexing, an FMCW chirp, or an adaptive non-uniform sampling strategy. With frequency multiplexing, one can first use lower ultrasound frequencies that suffer less attenuation to obtain a coarse reconstruction, and then bootstrap that estimate with higher-frequency data to improve the resolution - with multiple receivers this could be done simultaneously. With FMCW one can use short-pulse echoes similarly to echolocation by bats. Many bat species emit long, sweeping calls that change frequency over time, then shape those calls into brief pulses. By doing so, they achieve both fine-range discrimination (thanks to the wide frequency sweep) and precise spatial localization (due to the pulse’s time-localization). In our NLoS system, one could drive the transducer with a similar chirped burst-sweeping, for example, from 50 to 100 kHz over a few hundred microseconds and then apply matched-filtering (which is similar to lock-in techniques) on reception to compress the return into a sharp, high-signal-to-noise envelope. Finally, adaptive sampling could dramatically reduce acquisition time by using a preliminary reconstruction to guide subsequent measurements toward regions with the greatest detail. One could then employ advanced inference techniques, such as kernel-density estimation, Gaussian-process regression, or artificial neural networks to predict both optimal sampling locations and the underlying spatial structure of the scene. Prior work has demonstrated that ANNs can reconstruct spatial features from a single time trace^29^. By integrating wave-based reconstruction with these machine-learning or statistical methods, we can minimize the number of required scan positions and dramatically accelerate image acquisition.

Another way to improve the spatial resolution is to harness non-linear acoustic mixing or specifically, sum-frequency generation. Although this requires driving the transducer at elevated power levels, the resulting higher-frequency component would deliver substantially finer detail.

We believe that together, these enhancements pave the way for more practical and versatile NLOS imaging systems. Beyond hidden-scene imaging, our full-wave, phase-sensitive reconstruction approach can also benefit fields like medical diagnostics, robotics, surveillance, and autonomous navigation: in all these cases, full-wave reconstruction can deliver millimeter-scale detail in three-dimensional reconstructions irrespective of the acquisition scheme.

## Methods

### Setup description

**Fig. 5:**
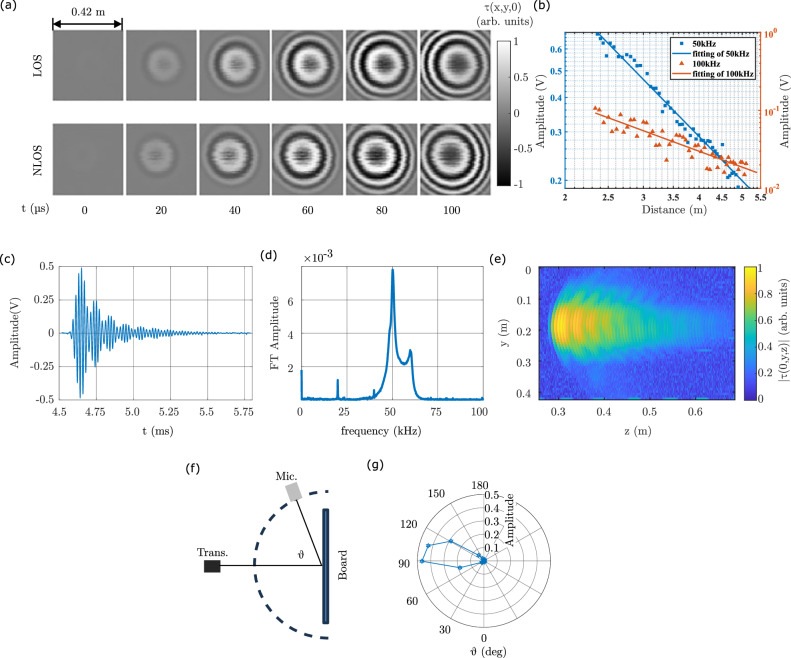
Experiment details. **a** *E*(*x*, *y*, *t*) for line-of-sight and in reflection from the relay surface. In both cases the transducer was fixed and the microphone performed a scan at a 1.6 m distance from the transducer. **b** Shows the measurement of the signal falloff, where the transducer and microphone are fixed and a flat metal board is moved away from the setup. The points represent the maximum values of the mean obtained from 20 acquisitions, and the decay follows an approximate power-law behavior of *d*^−1.6^ for 50 kHz transducer and *d*^−2.1^ for 100 kHz transducer. **c** Shows the typical time dynamics of the microphone signal *E*(0, 0, *t*). **d** Is the Fourier transform of the time trace in (**c**), showing the spectral content of the transducer signal. **e** Is the cross-section of the ultrasound signal datacube across the *x* plane. **f** The setup used to assess the specularity of typical surface: the transducer is fixed along the axis perpendicular to the target plane, while a microphone moves in a semicircular path, recording signal attenuation. **g** The polar plot showing the reflectivity pattern. Most of the signal is concentrated within  ±20°, which is similar to the transducer divergence, indicating mostly specular reflection.

The setup is illustrated in Fig. 1. The transducer (Unictron-A50A 50 kHz or Unictron-A100A1 100 kHz) is fixed together with the ultrasound microphone (Avisoft Bioacoustics CM16-CMPA, Frequency range 2–200 kHz) on a custom-built XY scanning stage that faces a plasterboard wall. The transducer is driven by a function generator and a custom-built amplifier, operating at a peak-to-peak voltage of 400 V. The return signal is recorded using a National Instruments Analog to Digital Converter (NI6310) typically at a sampling rate of 1 MS/s. The imaging model in Eq. (1) depends on the function *E*(*x*, *y*, *z*, *t*), which is determined by the transducer characteristics. This function can be written as

2 $$E(x,y,z,t)=D(x,y,z){e}^{-i[kz+\phi (x,y)]}F(t)$$

where *D*(*x*, *y*, *z*) determines the directionality of the transducer, *k* is the wavevector, *ϕ*(*x*, *y*) is the phase of the ultrasound signal, and *F*(*t*) is determined by the spectral response of the transducer. Information about *D* can be obtained by scanning the microphone in a certain plane when facing the transducer, which is fixed, and recording the ultrasound signal. In Fig. 5a, we show the results of such a measurement at 1.6 m distance between the transducer and the microphone scan plane, indicating that *E* is a bell-shaped function with a half-width of approximately 50 cm. In the bottom panel of this figure, we show the same measurement but in reflection from the wall (at the same scan-plane to object distance) showing very similar dynamics, which indicates that the reflection from the wall is mostly specular. In Fig. 5c, we show the typical time trace *F*(*t*) of the microphone signal, and the spectrum is shown in Fig. 5d. The full spatio-temporal profile is obtained by recording the time traces whilst scanning in x-y coordinates and shows some space-time coupling the transducer signal is extended in time. Figure 5e shows the cross-section of the ultrasound signal datacube in the x-t plane, from which *ϕ*(*x*, *y*) can be estimated.

In order to estimate the attenuation and thus the *α* factor in Eq. (1), we recorded the relative intensity of the return signal for a plane object as it was moved away from the scan plane, (in reflection from the wall). The results of this measurement are shown in Fig. 5b: for a 50 kHz transducer, the decay follows approximately d^−1.6^, while for a 100 kHz transducer, it is approximately d^−2.1^. These decay rates can be explained by two factors: first the ultrasound signal is attenuated with distance and the attenuation coefficient is proportional to the square of the sound frequency^30^, which is supported by the fact that we get higher decay rate at 100 KHz. The other factor is a small diffuse reflection from the surfaces. In Fig. 5g we show a measurement of the reflection directionality for a cardboard plate in a setup shown in Fig. 5f. Reflection is observed within an angle of approximately 40°, which adds to energy losses and contributes to the decay of the return signal with distance.

### Data processing

Data processing followed three steps. First, we interpolated the data cube in the spatial domain, the x-y plane visibly enhancing the spatial resolution. As previously mentioned, the resolution is determined by the ultrasound wavelength and the dimension of the synthetic aperture. The number of points—55 × 55 and synthetic aperture dimension define the spatial sampling. To fully resolve the maximum possible resolution, the sampling should be finer than or equal to the diffraction limit^5^. For our system, this would require a step size of less than 5 mm, corresponding to a sampling grid of over 100 × 100 points. However, the scan time increases by a factor of *N*^2^, where *N* is the number of spatial points per axis. We found that, even in this moderately undersampled regime, bicubic (2x) interpolation before image reconstruction, can achieve resolution at the diffraction limit while being 4 times faster in acquisition time as compared to the higher sampled case.

Second, in order to decrease data size, we downsampled the original 1 MHz sampling rate in the time dimension. For the 50 kHz transducer, the sampling rate was reduced to 125 kHz, and for the 100 kHz transducer, the sampling rate was downsampled to 250 kHz, with an oversampling rate of 1.25.

Third, we processed the data in the frequency domain using a band-pass filter, retaining only the signals in the range of  ±30 kHz from the center frequency of the ultrasonic transducer to improve the signal-to-noise ratio and to remove interference from uncorrelated frequencies.

## Supplementary information

Supplementary information

## Data Availability

The data generated for this work are available at 10.5525/gla.researchdata.2046.

## Code availability

The code written for this work is available at 10.5525/gla.researchdata.2046.
