# Supplementary material for: Ultrasound synthetic aperture non-line-of-sight imaging
Source: Commun Phys. 2025 Nov 17;8(1):432. doi: 10.1038/s42005-025-02335-3 (PMC12642816; doi:10.1038/s42005-025-02335-3)
Supplement: Supplementary file 1 — Supplementary information [file 42005_2025_2335_MOESM1_ESM.pdf]

Ultrasound Synthetic Aperture Non-line-of-Sight Imaging (Supplementary Information)

Tailin Li[1,3,4,5], Ilya Starshynov[2], Khaled Kassem[2], Zongliang Xie[1,3,4,5], Ge Ren[1,3,4,5], Yihan Luo[1,3,4,5], Daniele Faccio[21,*]

[11] *National Key Laboratory of Optical Field Manipulation Science and Technology, Chinese Academy of Science, Chengdu 610209, China*
[2]*School of Physics & Astronomy, University of Glasgow, G12 8QQ Glasgow, UK*
[3] *Key Laboratory of Optical Engineering, Chinese Academy of Science, Chengdu 610209, China*
[4] *Institute of Optics and Electronics, Chinese Academy of Science, Chengdu 610209, China*
[5] *University of Chinese Academy of Science, Beijing 100049, China*

SUPPLEMENTARY NOTE 1: RESOLUTION ASSESSMENT

Here we show that the resolution of our technique is identical to that of conventional line-of-sight imaging. Let's consider the process of retrieving an image using the f-k migration algorithm. Suppose a point source at $\mathbf{r_0} = (x_0, y_0, z_0)$ emits a spherical wave,

$$E(\mathbf{r}, t) = \frac{1}{4\pi} \frac{\delta\Big(t - \frac{\|\mathbf{r}-\mathbf{r}_0\|}{v}\Big)}{\|\mathbf{r} - \mathbf{r}_0\|}, \tag{S1}$$

where v is the speed of sound and $\mathbf{r} = (x, y, z)$. This wave is recorded at a plane $z = 0$, and the Fourier transform of this recording $E(x, y, 0, t)$ is:

$$\mathcal{F}\{E\}(k_x, k_y, \omega) = \frac{i}{2\alpha} e^{-i\left(k_x x_0 + k_y y_0 - \alpha z_0\right)}, \quad \alpha = \sqrt{\left(\frac{\omega}{v}\right)^2 - k_x^2 - k_y^2}, \quad \omega > 0, \quad \Im(\alpha) > 0. \tag{S2}$$

Now the Stolt interpolation presumes a change of variables $\omega \to v\sqrt{k_x^2 + k_y^2 + k_z'^2}$, so that $\alpha = k_z'$, which leads to

$$\mathcal{F}\{E\}(k_x, k_y, k_z') = \mathcal{F}\{E\}(k_x, k_y, \omega(k_z')) \frac{d\omega}{dk_z'} = i\, v\, e^{-i\,(k_x x_0 + k_y y_0)} \frac{e^{\,i\, k_z'\, z_0}}{2\sqrt{k_x^2 + k_y^2 + k_z'^2}}, \tag{S3}$$

and upon taking an inverse Fourier transform of this expression we recover the point source at $\mathbf{r_0}$:

$$E(\mathbf{r}, t = 0) = \delta\Big(\|\mathbf{r} - \mathbf{r}_0\|\Big). \tag{S4}$$

In practice, however the finite square collection aperture of side D at a distance L from the object means only spatial frequencies up to

$$k_{x,y}^{max} = \frac{\omega}{v} \sin\theta_{max}, \quad \sin\theta_{max} = \frac{D/2}{\sqrt{L^2 + (D/2)^2}}, \tag{S5}$$

are sampled, where $\sin\theta_{max}$ is the numerical aperture, NA, of the system. Thus the minimal resolvable feature in the image is:

$$\delta x/y_{min} = \frac{\pi}{k_{x,y}^{max}} = \frac{\lambda}{2\text{NA}} \tag{S6}$$

* daniele.faccio@glasgow.ac.uk

This expression also provides an estimate of how the resolution would scale with L. The axial (depth) resolution is determined by the finite pulse bandwidth. A pulse of linewidth $\Delta\omega$ spans a k_z range $\Delta k_z = \Delta\omega/v$ and thus

$$\delta z = \frac{\pi}{\Delta k_z} = \frac{\Delta\lambda}{2} = \frac{v}{2\Delta f} \tag{S7}$$

The best possible axial resolution is achieved when the probing pulse is a single cycle long, resulting in a bandwidth of $\Delta f = f$ and an axial resolution of $\delta z = v/2f$, which is 1.7 mm at 100 KHz and 3.4 mm at 50 KHz.

In conventional optical NLOS imaging the expressions for lateral and axial resolutions have similar form:

$$\delta x = \frac{c \times \text{FWHM}}{2\text{NA}}, \quad \delta z = \frac{c \times \text{FWHM}}{2}, \tag{S8}$$

where FWHM is the full width at half maximum of the time resolved photodetector. At $f = 100$ KHz, $\lambda = 3.43$ mm; thus, the FWHM of an optical system would need to be around 10 ps to achieve a similar resolution.

SUPPLEMENTARY NOTE 2: COMPARISON WITH OTHER TECHNIQUES

**Method**	**Modality**	**Resolution (lateral, axial, at a distance)**	**Limitations**
Kirmani et al. (2009) [1]	Femtosecond laser + picosecond sensor transient imaging	∼5 cm, ∼7.5 cm, at 15-20 cm	Proof-of-concept; requires ps timing; no full 3D
Velten et al. (2012) [2]	Femtosecond pulsed laser + streak camera ToF imaging	∼1 cm, 1 mm at 40 cm	Expensive ultrafast laser & streak camera; mechanical spot scanning; long acquisition times
Gupta et al. (2012) [3]	Pulsed laser + streak camera; diffuse-reflection tomography	1 cm, 0.5 mm at 30-80 cm	Low SNR from multiple scattering; needs fine time resolution; scene complexity sensitivity
Heide et al. (2013)∼[4]	Pulsed laser + SPAD camera; 2d scanning detector and laser	1-2 cm both at 1.5 + up to 0.8m	High computational and memory demands; sensitivity to calibration; ambiguities
O'Toole et al. (2018) [5]	Pulsed laser + confocal scan + SPAD; light-cone transform	∼1.25 cm, 0.9 cm at 0.64m	Raster scanning; limited FOV; moderate resolution gains
Lindell et al. (2019) [6]	Pulsed laser + SPAD array; wave-optics migration	1-2 cm, 8 mm at 1-1.5 m	High computational load; scanning (dense); high input optical power
Wang et al. (2021) [7]	Up-conversion single-photon detector + SPAD	2 mm, 180 µm at 63 cm	Limited FOV; specialized detector; low SNR (thus reflective target needed); low acquisition speed
Metzler et al. (2020) [8]	Speckle correlation + CNN	300 µm lateral at 1 m	Static scenes only; needs synthetic training; sensitive to model mismatch; limited FOV
Cao et al. (2022) [9]	Wavefront-shaping + single-pixel detector	0.6 mm lateral at 0.3 + 0.55 m	Wavefront-shaping needed; slow raster-scan; limited FOV; no 3D info; prior info about imaging plane needed
Willomitzer et al. (2021) [10]	Coherent dual-wavelength + holography	0.8 mm, 1-2 mm at 10 cm	Complex light source; narrow spectral tuning; speckle artifacts; high stability requirements
Thai et al. (2017)[11]	24 GHz radar multipath exploitation	∼1 m lateral, 18.75 cm axial at 2 m	Strong spatial ambiguities; coarse localization; low Doppler resolution; sensitive to scene model

Method	Modality	Resolution (lateral, axial, at a distance)	Limitations
Scheiner et al. (2020) [12]	FMCW Doppler radar	15 cm both at 5 m	Low spatial fidelity; clutter susceptibility
Tang et al. (2022)[13]	SFCW radar + custom multipath backprojection	28 cm both at ∼8 m	Specific geometry; low resolution; ghost artifacts
Aubry et al. (2021)[14]	FMCW radar + Reconfigurable Intelligent Surface	0.89° both, 10 MHz bw (15 cm, 2 cm, at 2 m)	Resolution depends on RIS size and beam steering; requires phase synchronization; complex RIS control
Wen et al. (2022) [15]	mmWave radar + inverse synthetic aperture (ISAR)	∼5.4 mm lateral, 3.8 cm axial at 2 m	Requires target motion; sensitivity to angular diversity; limited FOV; needs mirror model
Wei et al. (2022)[16]	mmWave radar + Backprojection	∼4 cm lateral, 4 cm axial at 0.6-0.8 m	Mechanical scanning; heavy processing; highly reflective (metal) relay wall
Lindell et al. (2019) [17]	Acoustic waves, FMCW preprocessing, deconvolution	10-15 cm, 10 cm at 2.5 m	Low resolution; heavy multipath; limited range; coherent artifacts; sparsity constrains
Cui et al. (2024) [18]	THz, SAR backprojection	10-15 cm, 5 mm, at 7 m	Mechanical scanning; sophisticated THz source and detector; multipath interference
Our work	Ultrasound, f-k migration	∼1 cm, ∼3 mm, at 1 m	Mechanical scanning; ghost artifacts

Supplementary Table 1: Comparison of our method with other NLOS modalities.

Over the past decade, NLOS imaging has evolved across various modalities, yielding significant improvements in spatial resolution and reconstruction range. Early optical approaches, such as Kirmani et al. (2009) [1], demonstrated the feasibility of transient imaging using femtosecond lasers and picosecond sensors, achieving lateral and axial resolutions of ∼5 cm and ∼7.5 cm at short distances (∼15-20 cm). Subsequent advances improved both resolution and depth: Velten et al. (2012) [2] and Gupta et al. (2012) [3] introduced streak cameras and confocal scanning, reaching millimeter axial resolution at distances up to 80 cm. The incorporation of SPAD detectors, and advanced reconstruction method such as light-cone transform and wave-optics migration (e.g., Heide et al. (2013) [4]; Lindell et al. (2019) [6]) further improved resolution to the 1-2 cm range laterally and sub-centimeter axially at meter-scale distances, albeit at the cost of high computational complexity and scanning time. More recent optical methods have pushed boundaries even further: Wang et al. (2021) [7] achieved ∼2 mm lateral and 180 µm axial resolution using up-conversion detection, while learning-based and wavefront-shaping approaches (Metzler (2020) [8]; Cao (2022) [9]) achieved sub-millimeter lateral resolution, though often in static, constrained scenarios without full 3D capability.

Parallel efforts in radar-based NLOS imaging explored the trade-off between longer working distances and coarser resolution. Early GHz-range radar methods (e.g., Thai et al. (2017) [11]; Scheiner et al. (2020) [12]) offered several meters of reach but suffered from meter- to decimeter-scale resolution. Later radar techniques, such as Wen et al. (2022) [15] and Aubry et al. (2021) [14], improved resolution to the millimeter scale laterally and centimeter axially by leveraging inverse synthetic aperture and reconfigurable surfaces, though often at the expense of increased system complexity or reliance on motion. In the acoustic domain, Lindell et al. (2019) [17] achieved ∼10-15 cm resolution at 2.5 m using FMCW and deconvolution. Current work using 50-100 KHz ultrasound and f-k migration significantly improves acoustic NLOS resolution to ∼1 cm laterally and ∼3 mm axially at 1 m, which is comparable to most optical NLOS techniques, but combining the benefits of practicality and high spatial fidelity.

SUPPLEMENTARY REFERENCES

[1] A. Kirmani, T. Hutchison, J. Davis, and R. Raskar, Looking around the corner using transient imaging, in *2009 IEEE 12th International Conference on Computer Vision* (2009) pp. 159–166.

[2] A. Velten, T. Willwacher, O. Gupta, A. Veeraraghavan, M. G. Bawendi, and R. Raskar, Recovering three-dimensional shape around a corner using ultrafast time-of-flight imaging, Nature communications **3**, 745 (2012).

[3] O. Gupta, T. Willwacher, A. Velten, A. Veeraraghavan, and R. Raskar, Reconstruction of hidden 3d shapes using diffuse reflections, Opt. Express **20**, 19096 (2012).

[4] F. Heide, M. O'Toole, K. Zang, D. B. Lindell, S. Diamond, and G. Wetzstein, Non-line-of-sight imaging with partial occluders and surface normals, ACM Trans. Graph. **38**, 10.1145/3269977 (2019).

[5] M. O'Toole, D. B. Lindell, and G. Wetzstein, Confocal non-line-of-sight imaging based on the light-cone transform, Nature **555**, 338 (2018).

[6] D. B. Lindell, G. Wetzstein, and M. O'Toole, Wave-based non-line-of-sight imaging using fast f-k migration, ACM Trans. Graph. **38**, 10.1145/3306346.3322937 (2019).

[7] B. Wang, M.-Y. Zheng, J.-J. Han, X. Huang, X.-P. Xie, F. Xu, Q. Zhang, and J.-W. Pan, Non-line-of-sight imaging with picosecond temporal resolution, Phys. Rev. Lett. **127**, 053602 (2021).

[8] C. A. Metzler, F. Heide, P. Rangarajan, M. M. Balaji, A. Viswanath, A. Veeraraghavan, and R. G. Baraniuk, Deep-inverse correlography: towards real-time high-resolution non-line-of-sight imaging, Optica **7**, 63 (2020).

[9] R. Cao, F. de Goumoens, B. Blochet, J. Xu, and C. Yang, High-resolution non-line-of-sight imaging employing active focusing, Nature Photonics **16**, 462 (2022).

[10] F. Willomitzer, P. V. Rangarajan, F. Li, M. M. Balaji, M. P. Christensen, and O. Cossairt, Fast non-line-of-sight imaging with high-resolution and wide field of view using synthetic wavelength holography, Nature Communications **12**, 6647 (2021).

[11] K.-P.-H. Thai, O. Rabaste, J. Bosse, D. Poullin, I. Hinostroza, T. Letertre, and T. Chonavel, Around-the-corner radar: Detection and localization of a target in non-line of sight, in *2017 IEEE Radar Conference (RadarConf)* (2017) pp. 0842–0847.

[12] N. Scheiner, F. Kraus, F. Wei, B. Phan, F. Mannan, N. Appenrodt, W. Ritter, J. Dickmann, K. Dietmayer, B. Sick, and F. Heide, Seeing around street corners: Non-line-of-sight detection and tracking in-the-wild using doppler radar, in *2020 IEEE/CVF Conference on Computer Vision and Pattern Recognition (CVPR)* (2020) pp. 2065–2074.

[13] Q. Tang, J. Li, L. Wang, Y. Jia, and G. Cui, Multipath imaging for nlos targets behind an l-shaped corner with single-channel uwb radar, IEEE Sensors Journal **22**, 1531 (2022).

[14] A. Aubry, A. De Maio, and M. Rosamilia, Reconfigurable intelligent surfaces for n-los radar surveillance, IEEE Transactions on Vehicular Technology **70**, 10735 (2021).

[15] Y. Wen, S. Wei, J. Wei, J. Liang, X. Zhang, and J. Shi, Non-line-of-sight imaging of hidden moving target using millimeter-wave inverse synthetic aperture radar, in *IGARSS 2022 - 2022 IEEE International Geoscience and Remote Sensing Symposium* (2022) pp. 555–558.

[16] S. Wei, J. Wei, X. Liu, M. Wang, S. Liu, F. Fan, X. Zhang, J. Shi, and G. Cui, Nonline-of-sight 3-d imaging using millimeter-wave radar, IEEE Transactions on Geoscience and Remote Sensing **60**, 1 (2022).

[17] D. B. Lindell, G. Wetzstein, V. Koltun, and I. C. Soc, Acoustic non-line-of-sight imaging, in *IEEE/CVF Conference on Computer Vision and Pattern Recognition (CVPR)*, IEEE Conference on Computer Vision and Pattern Recognition (2019) pp. 3773–6782.

[18] Y. Cui and G. C. Trichopoulos, Seeing around obstacles using active terahertz imaging, IEEE Transactions on Terahertz Science and Technology , 1 (2024).
